# Towards effective prevention and a complete cure for hepatitis

**DOI:** 10.1016/j.ebiom.2022.104156

**Published:** 2022-07-13

**Authors:** eBioMedicine

Every 30 seconds there is a person who dies of a hepatitis-related disease globally. WHO estimates that more than 354 million people are living with hepatitis, among whom hepatitis B (HBV) and hepatitis C (HCV) are responsible for 96% of liver cirrhosis, hepatocellular carcinoma, and liver transplantation, especially in Africa and the western Pacific region. Despite the fast development of vaccines and antiviral drugs, most countries are not on track to achieve the WHO target of eliminating viral hepatitis by 2030. The 2022 World Hepatitis Day, which takes place on July 28, calls on people across the world to raise awareness and take action against hepatitis.

Efforts towards eliminating viral hepatitis are particularly focused on HBV and HCV, owing to the high mortality and public health burden they represent. Combined efforts from scientists and physicians have made great progress in the development of effective prophylaxis and therapeutics against hepatitis. By the end of 2018, the HBV infant vaccine was introduced in 189 countries and regions. Meanwhile, novel direct-acting antiviral (DAA) drugs that target viral life cycles, such as viral entry, release, and replication, have emerged.

The persistent, covalently closed circular DNA (cccDNA) form of HBV is regarded as a target for HBV eradication. However, none of the available drugs can inhibit the transcription of viral genes from cccDNA and genome-integrated DNA. In October, 2021, *Nature Medicine* published a phase 2 trial of bepirovirsen that targets all HBV messenger RNAs. The study showed acceptable safety, as well as a significant reduction of HBV surface antigen (HBsAg) in 31 patients with HBV infection. At the International Liver Congress 2022 (June 22–26, London, UK), GSK presented their updated data of bepirovirsen in patients with on and off stable nucleotide analogue therapy (abstract number LB004A). At the same meeting, Vir Biotechnology Inc (San Francisco, CA, USA) also updated their phase 2 trial data about VIR-2218, a small n-acetylgalactosamine interfering ribonucleic acid (siRNA), for the potential functional cure of chronic HBV infection (abstract number 654). Vir-2218 is designed to target the conserved X region of HBV so that it can silence all HBV transcripts, including cccDNA and integrated DNA of all ten HBV genotypes. A study published in *Nature Communications* in March, 2021, led by Alexander Ploss (Princeton University, NJ, USA), showed further details about how cccDNA is formed from relaxed circular DNA (rcDNA), indicating that the repair of minus or plus strand lesions of rcDNA require different set of DNA repair factors. More importantly, the study suggests that FEN-1 and RFC are the most rate-limiting factors in cccDNA formation, which could lead to new therapeutic targets for cccDNA elimination.

Gene-editing approaches have also attracted wide interest for targeting integrated HBV and cccDNA in a sequence-specific manner. According to the preclinical data published in May in *Molecular Therapy*, an engineered ARCUS nuclease developed by Precision BioSciences (Durham, NC, USA) efficiently targeted and degraded 85% of the cccDNA in mouse and non-human primate models. Another study (*Mol Ther Methods Clin Dev*) led by Keith Jerome (Fred Hutchinson Cancer Research Center, WA, USA) examined the safety of CRISPR-Cas9-based therapy in mouse models. In their study, HBV-specific AAV-SaCas9 therapy was well tolerated and efficiently decreased total liver HBV DNA and cccDNA. Further optimisation of gene editing approaches could bring us closer to a cure for HBV infections.

The antiviral therapy of HCV is much more efficient compared with those available for HBV. Specific DAA treatment regimens have been approved as first-line therapy for each HCV genotype. However, very few HCV vaccines have progressed to clinical trials owing to the high genetic diversity of HCV. A randomised trial (*N Engl J Med*) published in February, 2021, led by Andrea Cox (Johns Hopkins University School of Medicine, MD, USA) evaluated the safety and efficacy of a recombinant chimpanzee adenovirus 3 vector priming vaccination followed by a recombinant modified vaccinia Ankara boost. Although the vaccine could elicit T-cell responses against HCV proteins, it unfortunately failed to lower the incidence of chronic HCV infection compared with placebo. To accelerate the research in HCV vaccines, the National Institute of Allergy and Infectious Diseases hosted a symposium (Vaccines against hepatitis C virus: Harnessing Immunity to Eliminate Global Hepatitis C) in March, 2022, on the latest research related to HCV vaccine development. New findings in HCV immunology and the emergence of new vaccine platforms, such as mRNA, were discussed. In particular, Mansun Law from Scripps Institute (CA, USA) introduced the challenges in developing vaccine antigens based on HCV envelope glycoproteins E1E2. Bali Pulendran at Stanford University (CA, USA) shared his thoughts about rapid assessment of HCV vaccine candidates that are inspired by the development of Pfizer BNT162b2 mRNA vaccine. Although obstacles remain, scientists look forward to the success of HCV vaccines in the next decade.

Fewer than 10 years remain to reach the WHO goal of hepatitis elimination. As the world tries to recover from the COVID pandemic, a mysterious hepatitis has suddenly swept across many countries. As of June 7, 2022, more than 700 paediatric cases of an unexplained acute hepatitis have been reported since the first case appeared in the UK. Laboratory tests have ruled out all known hepatitis viruses. The potential association between adenovirus and the disease, which has been widely discussed in hospitals in the UK, is under investigation. Other risk factors, such as current or previous infection with SARS-COV-2, or side-effects of SARS-CoV-2 vaccines, are also being examined. So far, none of these hypotheses can fully explain the severe clinical manifestations of this acute hepatitis in children. Interestingly, contrary to reports from the UK, a survey published in June (*MMWR Morb Mortal Wkly Rep*) led by Anita Kambhampati of the US Centers for Disease Control and Prevention (GA, USA) showed no increase in the number of hepatitis-related emergency department visits or hospitalisation between Oct, 2021, and March, 2022, among children aged between 0 and 11 years, compared with pre-COVID-19 pandemic levels. Thus, whether the cases of unexplained hepatitis represent a new type of hepatitis or only one unidentified hepatitis type that has long existed, remains controversial. Now the outbreak is under close monitoring.

Regardless of impressive efforts made in prevention and treatment, major challenges still stand in the way of the goal of hepatitis elimination. In addition to drug development, attention also needs to be paid to inequities in health-care delivery, which is crucial for early diagnosis and treatment of hepatitis. *eBioMedicine*, as part of *The Lancet Discovery Science*, continues to serve as a publishing platform for translational studies about human diseases, including hepatitis.

